# Acute effects of foam rolling and dynamic stretching on angle-specific change of direction ability, flexibility and reactive strength in male basketball players

**DOI:** 10.5114/biolsport.2023.121325

**Published:** 2022-12-13

**Authors:** Haoxiang Yuan, Junjie Mao, Canfeng Lai, Haiping Lu, Yadan Xue, Qingshan Liu

**Affiliations:** 1 School of Physical Education, Hangzhou Normal University, Hangzhou, 311121, China

**Keywords:** Warm-up protocol, 505 agility, Y-shaped agility, Change of direction deficit, Reactive strength index, Sit and reach, College basketball player

## Abstract

The purpose of our study was to determine the acute effects of dynamic stretching (DS), foam rolling (FR) and foam rolling combined with dynamic stretching (Combo) protocols on angle-specific change of direction (COD) ability, drop jump (DJ) performance and flexibility. Using a counterbalance crossover study design, eleven male basketball collegiate players (20.7 ± 0.6 years) were randomly assigned to one of the four protocols – control (CON), DS, FR, Combo – for each session, for a total of four sessions. A more aggressive foam cylinder with raised nodules, which is thought to be effective in stimulating the deep layer of muscle tissue, was used to observe for changes in their performance during sit and reach (SAR), DJ and COD tasks in 45 and 180 degrees. One-way repeated measures ANOVA was used to identify differences of each variable separately between interventions. The SAR after three interventions compared to the CON was significantly improved (F _(3,30)_ = 5.903, P = 0.003, η^2^ = 0.371). In the 505 test, both limbs failed to show a significant improvement in COD deficit. The non-dominant limb showed a significant improvement of 6.4% after FR when performing the Y-shaped agility (F _(3,30)_ = 4.962, P = 0.0065 < 0.05, η^2^ = 0.332). In the DJ, the reactive strength index and contact time changed significantly by 17.5% and -17.5% (η^2^ = 0.518, η^2^ = 0.571), respectively, immediately after FR. The current research suggested that FR may have an enhancing effect on COD speed in a 45° cutting task and neuromuscular function, while having the potential to improve non-dominant limb deficits in both COD tasks. In contrast, the Combo warm-up protocol did not produce a cumulative effect, suggesting the need for coaches to remain cautious about excessive warm-up duration.

## INTRODUCTION

Massage therapy for the relief of myofascial pain syndrome and muscle dysfunction can be traced back thousands of years [1, 2]. Self-myofascial release (SMR) is a form of manual massage. The foam roller as an SMR tool with its convenient, low-cost and easy-to-use features has been favoured by coaches and sports enthusiasts in recent years, widely used in training practice and scientific research fields [3]. Foam rolling (FR) can alter local blood flow volume and neuromuscular excitability, break up trigger points, improve muscle-tendon unit compliance, lower the pain perception threshold, and promote exercise-induced muscle damage (EIMD) recovery [4]. These physical and psychological changes acutely affect athletic performance [5].

Many researchers have evaluated the acute effects of FR or DS alone on flexibility by various methods [6–10]. Although moderators of the treatment (type of roller, rolling speed, duration and muscle) or dynamic movements used vary, most studies found strong improvement in joint range of motion (ROM) [6–9]. However, limited knowledge is available on the combined treatment of FR and DS (Combo), especially on the acute effects on ROM and performance [11]. A review by Anderson et al. [12] that included only four studies compared the effects of FR with combined treatment on ROM and performance-related variables. They concluded that the combination programme seemed to produce the greatest gains in jump power [13–15] and agility [14, 16] but had little effect on ROM [14, 16] compared with stretching alone. Similar performance enhancements were also observed in a quantitative review by Konrad et al. [11], but they concluded that FR followed by stretching led to a significant overall effect on ROM compared with the control group. The inconsistent findings and limited evidence of the Combo protocol led us to further investigate whether there existed a cumulative effect.

Changes in tissue properties and neuromuscular function after FR offer the potential for enhanced sports performance [17], particularly in basketball, which involves a lot of jumping and change of direction (COD) tasks with fast stretch-shortening cycles (SSC). The drop jump (DJ) test can be used to identify reactive strength as a tool to examine neuromuscular function [18]. In a laboratory study, Bradbury-Squires et al. found that FR effectively enhanced individuals’ neuromuscular efficiency during a lunge [19]. In the field test, Wang et al. similarly found a significant increase in the reactive strength index using vibration FR in the DJ test [20]. However, Richman et al. used the DJ and found no significant change in jump height in the Combo group compared to DS alone. Due to the lack of reported contact time, these findings leave researchers with difficulty in determining the detailed impact of treatment on neuromuscular function.

In exploring the acute effects of FR on agility, Peacock et al. used the pro agility test and found a significant decrease in completion time [14]. A similar effect was demonstrated in the Edgren 10 s side step test [21]; however, no significant changes were observed when assessed by Richman et al. [13] and Lopez-Samanes et al. [10] using the T test. The conflicting results in these agility tests may be attributed to differences in the number of directional changes, cutting angle, acceleration distance, entry velocity, etc. COD ability reflects the underlying ability of an individual to rapidly decelerate, change movement patterns, and re-accelerate processes [22]. It is a key component in basketball that can distinguish between players at different levels and positions. [23]. Therefore, COD should be assessed using an independent testing protocol rather than an agility test with multiple COD tasks [24]. Furthermore, recent studies have shown that in performing COD tasks, those below 90 degrees are velocityoriented and those above 90 degrees are strength-oriented, suggesting specificity in the physical and biomechanical demands of athletes for COD tasks with different angle and entry speeds [25]. To better explain the conflicting results of the acute effects of FR on agility, our study was designed to observe athletic performance with a single, unilateral, angle-specific COD task at the same entry velocity. The aims of our current study were: 1) to investigate the acute effects of deep tissue foam rolling on specific angle COD ability, drop jump performance and flexibility in male basketball players; and 2) to explore the cumulative effects of deep tissue foam rolling combined with dynamic stretching on drop jump performance and flexibility.

## MATERIALS AND METHODS

Although foam rolling as a pre-exercise method has been extensively applied by many coaches, the findings on its acute effects of performance variables are not consistent [5]. In this study, a more aggressive foam cylinder with raised nodules, which is thought to be effective in stimulating the deep layer of muscle tissue [26], was used to explore the effects on COD ability, reactive strength index and flexibility. A random crossover design was used to compare selected warm-up protocols (control, DS, FR, Combo). The intervention sequence for each player was randomly generated (https://www.randomizer.org) and concealed until interventions were assigned. To better control environmental and personal state variations, each participant performed four sessions with 48 h intervals at the same time of the day. The venue chosen was a gymnasium with a wooden floor at Hangzhou Normal University (temperature: 19.5 ± 0.9°C; humidity: 53 ± 2.2%; TH-007, SanLiang, Japan). The intervention protocol in the current experiment was modified from Peacock’s study to maintain consistency of intervention doses as well as to facilitate comparative studies [14]. To our knowledge, no research exists that has used this particular type of foam roller to assess its effect on COD ability at different angles. Thus, the approach to this problem was to compare deep tissue foam rolling, dynamic stretch, and the combined protocol on Y-shaped agility, 505 test, drop jump and sit and reach test (SAR).

### Subjects

To decrease expectancy effects, the participants in our research did not use foam rolling as a pre-exercise method. The subjects were collegiate male basketball player from the Sports Department of Hangzhou Normal University in the off-season (2 times/week, low to moderate intensity), with 11 players recruited (age: 20.7 ± 0.6 years; height: 181.8 ± 5.8 cm; body mass: 74.4 ± 9.4 kg; BMI: 22.4 ± 1.7 kg/m^2^). The athletes were informed in advance of the injury risks and the purpose of the study, which was approved by the Institutional Review Board of Hangzhou Normal University. The authorized informed consent was signed voluntarily. In addition, subjects also needed to fill in the injury history questionnaire to confirm their eligibility before the experiment, and they were included in the experiment according to the standard after strict screening. Inclusion criteria: (a) aged between 18 and 24; (b) have been engaged in sports for at least three years; (c) keep daily physical activity. Exclusion criteria: (a) history of major lower limb injuries within the past three years; (b) participated in the latest regular stretching training programmes; (c) neuromusculoskeletal or cardiovascular disease in the past year that may potentially affect speed, strength, explosive power and other qualities; (d) during the test period, any injured participant would automatically quit the experiment process. A coach certified by the National Strength and Conditioning Association (NSCA) administered the entire experimental protocol to ensure proper exercise execution, providing the subjects with technical support and verbal encouragement.

### Testing Procedures

**FIG. 1:**
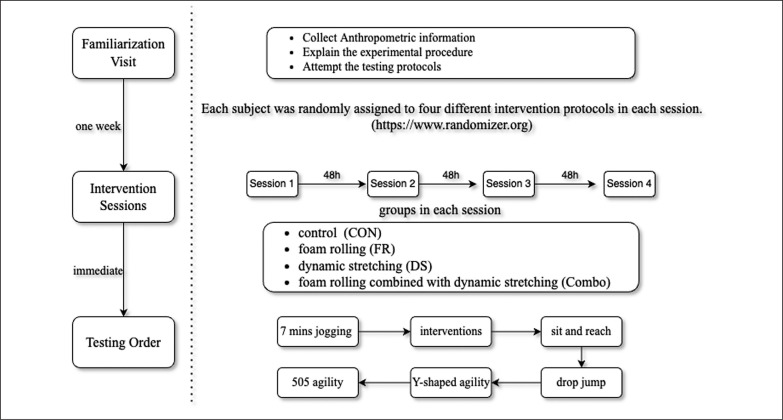
Experiment flowchart.

For the familiarization visit, a well-trained assistant collected height, weight, BMI (InBody J30, Biospace, Seoul, Korea), leg length and a questionnaire with basic information of each player (Figure 1). The leg length was defined as the length of the anterior superior iliac spine of the right lower limb to the distal end of the medial malleolus [27]. Subjects were familiar with the standardized warm-up movements and testing protocols under the guidance of professional staff. Each participant randomly completed the control (CON), DS, FR and Combo protocols in four sessions at 48 h intervals. The test sequence for each session was SAR, drop jump, Y-shaped agility test and 505 agility test. Before the interventions and CON, players had a jog with 60–75% of maximal heart rate for 7 minutes (Polar H10, Polar Electro Oy). The maximum heart rate was estimated by the following equation: HR_max_ = 208-(0.7*age) [28]. During the experiment, athletes were asked to avoid caffeine intake and strenuous exercise while maintaining a routine of physical activity and training. All tests are completed within 30 min to avoid attenuating the effects induced by the intervention [6, 29].

### Intervention warm-up protocols

For self-myofascial release session, the foam roller selected in this experiment is a hollow roller (Joinfit, Suzhou Jiayou sports and leisure products Co., Ltd) with a convex surface, size 42 × 14 cm, material EVA outer layer +PVC inner tube. The FR protocol was adapted from Peacock et al. [14] including the legs (gastrocnemius, soleus), hamstrings (semitendinosus, semimembranosus, biceps femoris), quadriceps femoris, hips (gluteus maximus, gluteus medius, gluteus minimus), and back of the chest (erector spine, multifidus). The current study used a metronome to assist athletes to keep rolling uniformly at a speed of 6 seconds/back and forth. The continuous stimulation time of each unilateral muscle group was 30 seconds. There were 2 sets with an interval of 2 minutes.

The dynamic stretching programme is based on Peacock et al. [14] and incorporates parts of the F11+ warm-up programme [30], including mobility (body-weight squats, bilateral lunges, squat jumps) and flow manoeuvres (lunges and arm rotations during march, single leg Romania deadlift, high knees, back kicks during march, stride run). Each drill was performed for two sets of ten (meters/ repetitions), with 2-minute intervals. The professional staff supervised the entire process.

The combination warm-up programme (Combo) incorporates foam rolling followed by dynamic stretching. The details are as described above, with each part completing one set.

### Sit and reach

The SAR is a valid and reliable (ICC = 0.92) test to appraise the flexibility of the posterior chain muscle groups [31]. Subjects were asked to remove their shoes and sit on the floor with their back against the wall; place both feet flat against the SAR box (ZW, Shanghai yilian, Inc, China) with the knees extended; slowly slide the module forward as far as possible with both fingertips and avoid jerky movements under supervision by an assistant. The SAR box uses the level of the feet as the zero mark. The score is recorded to the nearest centimetre on the reach indicator. Two attempts were measured with the mean used for analysis.

### Drop jump

The reactive strength index (RSI) identifies the individual’s ability to transition from eccentric contraction to concentric movement, as measured by the drop jump test in our study. The reliability and validity of the software (My Jump 2, iPhone 11, IOS 15.0 version) have been strictly verified and it has been promoted in field-based research and training practice recently due to its characteristics of high portability, simple operation and low cost [32]. RSI = flight time/contact time. Athletes stand on the 30 cm jump box with their hands on their hips to avoid an arm swing. The assistant reads out two instructions: (a) Jump with all your might; (b) Minimize the contact time. Three attempts were completed with 30 s recovery between jumps and the best RSI was recorded.

### 505 agility

As previously mentioned, because of its ability to differentiate unilateral turning ability, the 505 test was included in the present research. For the 505 test an established method was used, with a valid and reliable mobile app (COD Timer, iPhone 11, IOS 15.0 version) to record time [33]. A previous study showed that the change of direction deficit (CODD) in the 505 test is more effective in reflecting an athlete’s ability to change direction [34]; thus the best CODD of two trials was used for the analysis for each leg with 2-minute intervals. Time was recorded to the nearest 0.001 seconds. If the subject changed direction before hitting the turning point, or turned off the incorrect foot, the trial would be disregarded and another trial completed.

### Y-shaped agility

**FIG. 2:**
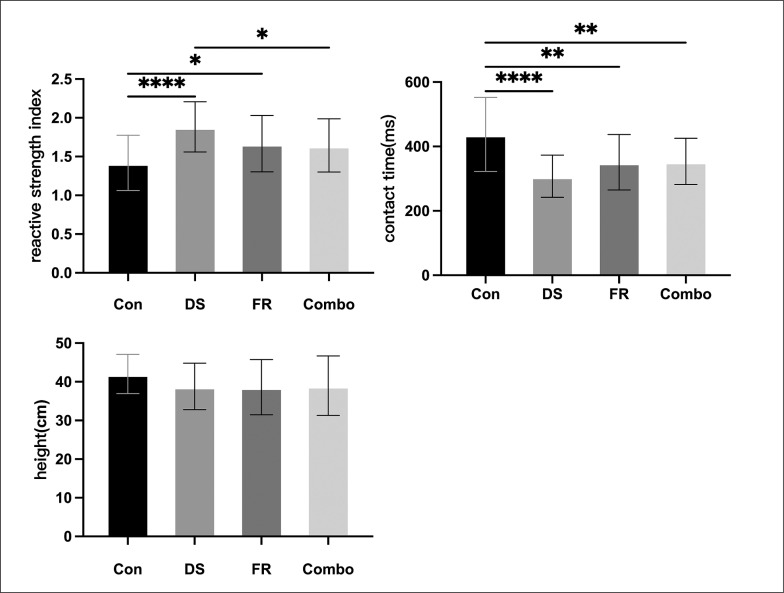
The acute effects of different warm-up protocols on the drop jump performance.

The current Y-shaped agility test modified from a previous study [35] added a 5 m acceleration zone to obtain a similar entry velocity in the COD task (Figure 2). The best of three trials with 2 minutes for each direction was recorded by a timing system (J14D-3R, Ziyu Electronic Technology, China). The change of direction speed (CODS) was used for analysis.

In our research, we define the dominant leg by the better performance in the control group for each angle-specific COD task.

### Statistical Analyses

All data were analysed using IBM SPSS Statistics Version 21.0 (IBM Corporation, Armonk, NY, USA), and the main descriptive parameters were calculated. The Shapiro-Wilk test was used to verify variables for normality. To determine the statistical difference of differences among the CON, DS, FR and Combo groups, repeated-measures analyses of variance (ANOVAs) were conducted. Data that did not meet Mauchly’s sphericity test hypothesis were corrected using Greenhouse-Geisser correction. Multiple comparison analyses and graph drawing were examined using Bonferroni post hoc measures by GraphPad Prism 9. The alpha (α) level was set to p ≤ 0.05 to determine significance.

## RESULTS

For the SAR test, one-way repeated measure ANOVA was used to determine the acute effects of different stretching interventions as warm-up methods on the performance of athletes in the SAR test; According to the Shapiro-Wilk test, the data followed a normal distribution. Mauchly’s sphericity assumption was satisfied (P = 0.723 > 0.05). Significant differences were found between the different warm-up protocols (F _(3,30)_ = 5.903, P = 0.003, η^2^ = 0.371). Based on the results of Bonferroni’s test, DS, FR and Combo protocols showed significant improvements in SAR compared to the CON (Cohen’s d = -0.397, -0.335, -0.327 respectively), but there was no statistically significant difference between the interventions (See Table 1).

**TABLE 1:** The acute effects of different warm-up protocols on the sit and reach test

Intervention	N	Mean (SD)	95% CI	P value
CON	11	14.24(5.63)	10.46–18.02	-
DS	11	16.37(5.26)	12.83–19.90	0.004*
FR	11	16.04(5.40)	12.41–19.66	0.019*
Combo	11	15.99(5.11)	12.56–19.42	0.023*

Note: CON, control group; DS, dynamic stretching group; FR, foam rolling group; Combo, foam rolling combined dynamic stretching group;

**FIG. 3:**
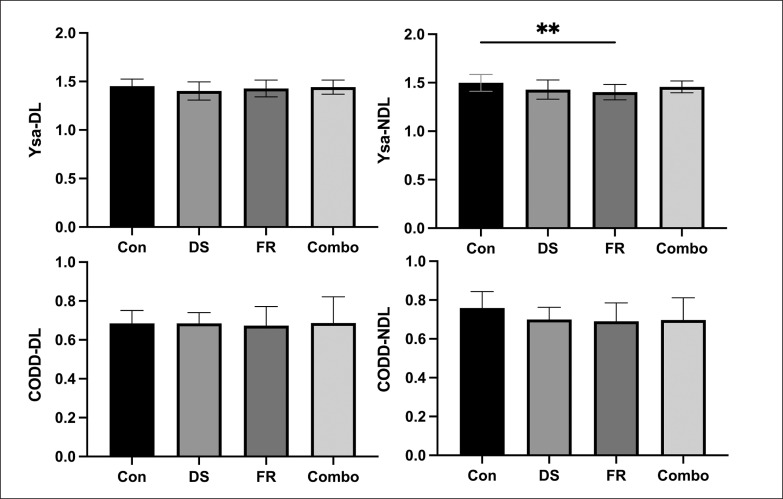
The acute effects of different warm-up protocols on the specific angle change of direction ability.

* , significant compared with the CON;

In the drop jump test, all parameters met Mauchly’s sphericity assumption. The athletes showed significant differences in RSI, CT (F_(3,30)_ = 10.738, p < 0.001, η^2^ = 0.518; F_(3,30)_ = 13.286, p < 0.001, η^2^ = 0.571) after different intervention protocols according to Table 2, but not in height (F_(3,30)_ = 2.353, p = 0.092 > 0.05, η^2^ = 0.190). The results of Bonferroni’s multiple comparisons are shown in Figure 3 and Table 2.

**TABLE 2:** The acute effects of different warm-up protocols on the drop jump performance

Indicators	Intervention	Mean	SD	95%CI	P value
RSI	CON	1.418	0.357	1.178–1.658	-
	DS	1.883	0.324	1.665–2.100	< 0.001*
	FR	1.666	0.363	1.423–1.910	0.030*
	Combo	1.644	0.342	1.414–1.873	0.040^*d^

CT (ms)	CON	437.5	115.3	360.0–514.9	-
	DS	307.5	65.63	263.4–351.6	< 0.001*
	FR	351.0	86.07	293.2–408.8	0.002*
	Combo	353.8	71.44	305.8–401.8	0.003*

Height(cm)	CON	42.02	5.073	38.61–45.42	-
	DS	38.80	6.010	34.76–42.83	0.231
	FR	38.62	7.123	33.84–43.41	0.178
	Combo	38.99	7.689	33.83–44.16	0.307

Note: RSI, reactive strength index;CT, contact time;CON, control group; DS, dynamic stretching group. FR, foam rolling group; Combo, foam rolling combined dynamic stretching group;

* , significant compared with the CON;

*d , significant compared with the DS; p value, compared with CON

For the Y-shaped agility test, there was no significant difference for the dominant leg in the time to complete the 45-degree change task between the baseline group and the groups after intervention, (F _(3,30)_ = 1.300, P = 0.293 > 0.05, η^2^ = 0.115), while the difference in CODS for the non-dominant limb was statistically significant (F _(3,30)_ = 4.962, P = 0.0065 < 0.05, η^2^ = 0.332). Bonferroni’s multiple comparisons test was conducted to compare the acute effects of each protocol. The non-dominant limb took significantly shorter time to complete the 45-degree COD after FR relative to the CON (Cohen’s = 1.157) (Table 3).

**TABLE 3:** The acute effects of different warm-up protocols on 45°change of direction task

Indicators	Intervention	Mean	SD	95%CI	P value
ysa-DL(s)	CON	1.453	0.072	1.404–1.501	-
	DS	1.402	0.094	1.339–1.466	0.431
	FR	1.428	0.086	1.370–1.486	> 0.999
	Combo	1.442	0.072	1.393–1.490	> 0.999

ysa-NDL(s)	CON	1.499	0.087	1.441–1.558	-
	DS	1.429	0.098	1.363–1.459	0.071
	FR	1.403	0.080	1.349–1.457	0.006*
	Combo	1.457	0.061	1.416–1.498	0.709

Note: CON, control group; DS, dynamic stretching group; FR, foam rolling group; Combo, foam rolling combined dynamic stretching group; ysa, y-shaped agility; NL, dominant leg; NDL, non-dominant leg;

* , Statistical significance compared to control group; P value, compared with CON

**FIG. 4:**
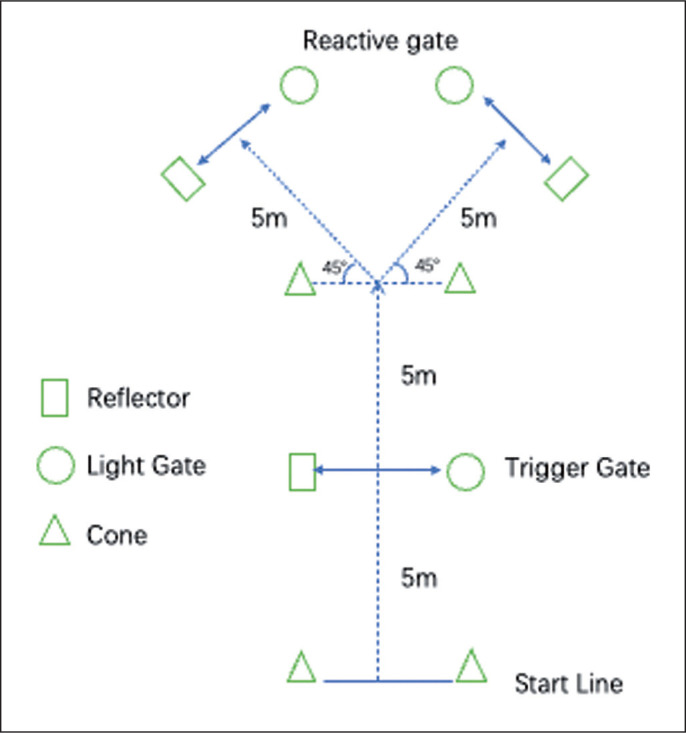
The modified Y-shaped agility test.

For the 505 test, because of the significant result of Mauchly’s test for sphericity (p = 0.026), Greenhouse-Geisser correction was used for statistical analysis of the CODD data for the dominant leg (F _(1.903, 19.03)_ = 0.055, P = 0.941, η^2^ = 0.005). There was no significant difference in the CODD of non-dominant limbs (F _(3, 30)_ = 1.527, P = 0.228, η^2^ = 0.132) among different intervention groups (Table 4 and Figure 4).

**TABLE 4:** The acute effects of different warm-up protocols on 180°change of direction task

Indicators	Intervention	Mean	SD	95%CI	Cohen’s d
CODD-DL	CON	0.686	0.066	0.6408–0.7301	-
	DS	0.685	0.055	0.6483–0.7221	0.003
	FR	0.673	0.099	0.6067–0.7396	0.131
	Combo	0.687	0.135	0.5967–0.7775	-0.017

CODD-NDL	CON	0.759	0.084	0.7028–0.8158	-
	DS	0.700	0.063	0.6577–0.7419	0.654
	FR	0.691	0.094	0.6273–0.7543	0.753
	Combo	0.697	0.115	0.6202–0.7743	0.682

Note: CON, control group; DS, dynamic stretching group; FR, foam rolling group; Combo, foam rolling combined dynamic stretching group; CODD, change of direction deficit; NL, dominant leg; NDL, non-dominant leg; Cohen’s d, compared with CON

## DISCUSSION

After undergoing warm-up protocols of DS, FR and Combo, male college basketball players significantly improved their SAR by 15.0%, 12.6%, and 12.3% compared with CON. A number of previous studies have found significant acute effects of FR or DS alone on improving joint mobility in athletes [5, 6, 8, 15, 26], which is consistent with the results of the present study. Stretching and FR enhanced ROM appear to have similar mechanisms related to decreased soft-tissue stiffness, increased stretch tolerance and/or thixotropic effects [17]. Since the treatment time was essentially the same as the duration of treatments in this experiment, it seems to explain the almost identical increase in DS and FR compared to the CON group (Cohen’s d = -0.397, -0.335 respectively). However, the Combo group was not significantly enhanced compared to DS or FR (Cohen’s d = 0.070, 0.008 respectively). Although other studies of combined protocols used knee joint ROM [8], hip flexion ROM [26], and sit and reach [15] in assessing flexibility, the results were also consistent with the present study. Anderson et al. concluded that the Combo protocol did not have a superior effect compared to DS alone [12]. Intriguingly, even when the DS was replaced by static stretching in the combined protocol, it did not show a greater additional effect [11]. In addition, a saturation effect may be responsible for the lack of superior effect of the combined protocol, which suggests that stretching or FR beyond a certain time results in a loss of flexibility gains [36]. The Combo protocol of this study led to a significant increase in flexibility compared to CON (Cohen’s d = -0.327), but did not show a cumulative effect, which may be related to the total duration of the intervention.

The results of the study showed that relative to the baseline group, the DS, FR, and Combo groups had improvements of the reactive strength index (RSI) by 32.8%, 17.5%, and 15.9% respectively, with the first two protocols showing statistically significant improvements. In this study, the 30-cm drop jump was used to assess the changes in the lower extremity reactive strength after different intervention protocols. This ability not only correlates with the speed of the speed-oriented COD task [25], but also reflects the changes in the neuromuscular control function of the athletes [18]. Specifically, the contact times were significantly shorter after three warm-up protocol (-29.7%, -19.7%, -19.1% respectively), while the jump heights were essentially the same as before (-9.4%, -8.1%, -7.2% respectively). This suggests that the increase in lower limb reactive strength may be a result of increased neuromuscular excitability, which led to completion of the stretch-shortening cycle (SSC) in a shorter period of time, resulting in a shorter contact time. Local compression rolling of the foam roller may alter muscle and tendon surface tension and mechanical stress, which in turn activates proprioceptors and chemoreceptors, triggering neural tissue excitation and reinforcing feedback mechanisms [37]. In the study by Werstein et al. [38], they compared the changes in RSI after the intervention in the control, static stretching and dynamic stretching groups. The results showed that dynamic stretching significantly improved the athletes’ RSI, which is consistent with our results. However, flight time was significantly improved while there was no significant difference in contact time, which is the opposite of the results of this study. This difference could be due to the different warm-up movements in the protocol. This study used four basic exercises, while the dynamic stretching in our experiment included squat jump, high knee, and running bounding to mobilize the organism more comprehensively. These forms of stretching actions may indirectly increase nervous system excitability [7]. FR may also increase the elastic potential energy of the musculotendinous unit during the eccentric phase at the completion of the SSC exercise by enhancing muscle compliance, leading to efficient performance during the concentric contraction phase [19].

Other studies [39] have suggested that foam rolling, when applied with pressure, decreases neuromuscular excitability and relaxes muscles, thus minimizing myofascial trigger point activity and pain. In a study by Godwin et al. [40], it was concluded that FR did not affect the performance of the drop jump. The control and experimental groups described in that experiment followed two protocols that used a basic warm-up, DS and a basic warm-up, FR, followed by DS, respectively. The two groups did not show significant differences in the results, probably because of the lack of a control group. Although our findings suggest that both DS and FR protocols led to a significant increase in RSI in athletes compared to the CON, the Combo group did not show a cumulative effect, which contradicts the results of a previous review [11]. After comparing the specific study protocols in this study with previous literature, it can be suggested that this may be due to fatigue of the athletes from the prolonged intervention protocol. Future studies need to further investigate the underlying physiological mechanisms of FR to enhance athletic performance and be alert to the detrimental effects of excessively prolonged warm-up activities on athletes’ physical performance.

The acute effects of the Combo protocol on the unilateral limb COD of athletes at specific angles were examined for the first time in our study, and the results showed a significant reduction in the 45-degree CODS of the non-dominant limb after FR. In the task of changing direction from different angles, athletes have different requirements in all aspects of sports quality [41]. Generally speaking, the task of changing direction exceeding 90 degrees is often considered as strength orientation, which means that the eccentric muscle strength showed a significant and large correlation with COD speed performance [42]. In the execution of a 180-degree turning task, it is more necessary for athletes to overcome deceleration at high speed with great eccentric strength. However, Madoni et al. found that FR did not seem to affect muscle eccentric hamstrings peak torque and EMG [43]. The results showed that the CODD of the dominant limb decreased by 0.04%, 1.79%, -0.23% respectively compared with the CON (0.686 ± 0.020 s) after three warm-up programmes, while that of the non-dominant side (0.759 ± 0.025 s) decreased by 7.84%, 9.02%, 8.17% and the differences were not statistically significant. Additionally, the process of re-acceleration is similar to the acceleration process of sprint. Richman et al. found no significant change when observing the acute effect of self-myofascial release on 10-yard short sprint [13], which may be another factor influencing the results of this study. The other type of COD task is the smaller angle (below 90-degree) COD task, which is speed oriented [25]. This type of COD task requires athletes to have good sprinting ability to maintain maximum speed through the COD as much as possible. In this study, it was found that after DS, FR, and Combo protocols, athletes’ CODS were shorter by 3.5%, 1.7%, 0.8% relative to the baseline (1.453 ± 0.072 s) for the dominant limb, and improved by 4.7%, 6.4%, and 2.8% for the non-dominant leg. Comparing the effects of FR on dominant and non-dominant limbs in different angular COD tasks, it was found that the enhancement effect was more pronounced in non-dominant limbs, suggesting that it may have a greater potential to improve lower limb asymmetry in the COD task. On the one hand, athletes are required to generate the maximum strength within 0.44–0.72 s of contact time in the turning step [24], while in the 45-degree cutting task, athletes’ contact time is shorter than in the 180-degree task [44]. Therefore, the framework of directional changing ability sub-quality established by Young et al. includes reaction strength [45]. In the 30 cm drop jump test of the current study, the results revealed a significant enhancement for RSI of the DS and FR groups. It proved that compared with the Combo group, both the two former groups showed a more obvious improvement, whether in the dominant limb or non-dominant limb, velocity-oriented or strength-oriented task, which is consistent with our COD test results. On the other hand, after warm-up, CODD of the non-dominant limb in the FR group 180-degree task was increased more than that in the DS group, which may be caused by the fact that dynamic stretching can significantly improve the sprint ability [7]. When completing the 180-degree COD task, the stronger the sprint ability, the faster the speed and the greater the momentum, the longer the deceleration time to zero is required [44]. However, some studies have shown that FR does not significantly affect athletes’ sprint ability, and may strengthen their reactive strength [6]. Therefore, the performance improvement after FR compared to DS may be more obvious when completing the 180-degree turning task. Unfortunately, at this stage of the study, it is not known why the difference in unilateral lower extremity CODS after FR in athletes exists.

Leaving aside the new findings, there are still some limitations in the current study. Firstly, the small sample size may limit the power of this study and increase the probability of false-positive results. The findings need to be interpreted with caution and a larger sample size study is expected to improve generalizability in the future. Due to the lack of experience with foam rolling and the use of a raised rigid rubber roller, some participants exhibited great discomfort, which would potentially enhance voluntary contraction and impact the true effect of the intervention. From this perspective, it suggested that the next stage needs to quantify pressure applied to the subject. Additionally, although reliable and valid software was used in this study to assess parameters related to the 505 and drop jump, more sophisticated instrumentation could be used in the future to capture more parameters (e.g., COD contact time, entry velocity) to help researchers better understand how treatment profoundly affects performance.

### Practical applications

For college men’s basketball players, warm-ups using DS, deep tissue FR and Combo protocols are effective in improving flexibility. However, the first two clearly maximize time efficiency. For small angle changes of direction drills or games involving fast SSC, pre-exercise FR is more recommended. Coaches need to be cautious in considering that the warm-up duration may not be too long.

## CONCLUSIONS

For collegiate male basketball players, both deep tissue FR and Combo pre-exercise protocols were effective in improving flexibility. Deep tissue FR had a positive effect on the reactive strength required in exercises involving SSC, which may result from enhanced neuromuscular function. For 180°, strength-oriented COD tasks, the FR failed to show better potentiation. In contrast, for the 45°, velocity-oriented COD task, the non-dominant limb showed a greater enhancement after FR, and it remains unclear why its effect on the bilateral lower limbs differed. The possible positive effects of FR on lower extremity asymmetry presented in motor tasks need to be explored further in future studies. Of particular note, the Combo protocol in this study did not produce a cumulative effect for all variables, and the specific threshold of the saturation effect needs more attention.

## Supplementary Material

Acute effects of foam rolling and dynamic stretching on angle-specific change of direction ability, flexibility and reactive strength in male basketball players
