# Supplementary material for: Acute effects of foam rolling and dynamic stretching on angle-specific change of direction ability, flexibility and reactive strength in male basketball players
Source: Biol Sport. 2022 Dec 13;40(3):877–87. doi: 10.5114/biolsport.2023.121325 (PMC10286619; doi:10.5114/biolsport.2023.121325)
Supplement: Acute effects of foam rolling and dynamic stretching on angle-specific change of direction ability, flexibility and reactive strength in male basketball players [file JBS-40-121325-s1.pdf]

SUPPLEMENTAL MATERIALS

TABLE. Effect size (Cohen's d) for outcome variables in post hoc comparisons.

Testing		SAR	Drop jump			505 agility		Y-shaped agility	
Effect size		Cohen's d	Cohen's d (RSI)	Cohen's d (CT)	Cohen's d (H)	Cohen's d (DL)	Cohen's d (NDL)	Cohen's d (DL)	Cohen's d (NDL)
CON	DS	-0.397*	-1.340*	1.498*	0.491	0.003	0.654	0.615	0.843
	FR	-0.335*	-0.716*	0.997*	0.518	0.131	0.753	0.301	1.157*
	Combo	-0.327*	-0.650	0.964*	0.461	-0.017	0.682	0.132	0.506
DS	FR	0.062	0.624	-0.501	0.027	0.128	0.099	-0.314	0.315
	Combo	0.070	0.690*	-0.533	-0.030	-0.020	0.028	-0.483	-0.337
FR	Combo	0.008	0.066	-0.032	-0.057	-0.148	-0.177	-0.169	-0.652

Note: *, statistical significance.

TABLE. Subject characteristics (N = 11).

Parameters	Mean ± SD
Age	20.7 ± 0.6
Body mass (kg)	74.4 ± 9.4
Height (cm)	181.8 ± 5.8
BMI (kg/m^2)	22.4 ± 1.7
Leg length (cm)	95.4 ± 4.1

Table. Control group test results in specific angle change tasks.

player	505 test				y-shaped agility test			
	change of direction deficit				change of direction speed			
	left leg	right leg	DL	ASI (%)	left leg	right leg	DL	ASI (%)
#1	0.629	0.683	left	-8.6	1.477	1.528	left	-3.5
#2	0.746	0.804	left	-7.8	1.427	1.474	left	-3.3
#3	0.758	0.804	left	-6.1	1.477	1.472	right	-0.3
#4	0.762	0.758	right	-0.5	1.402	1.339	right	-4.7
#5	0.675	0.638	right	-5.8	1.435	1.477	left	-2.9
#6	0.695	0.633	right	9.8	1.430	1.456	left	-1.8
#7	0.667	0.908	left	-36.1	1.562	1.594	left	-2.0
#8	0.583	0.759	left	-30.2	1.461	1.428	right	-2.3
#9	0.762	0.763	left	0.0	1.441	1.421	right	-1.4
#10	0.867	0.733	right	-18.3	1.594	1.716	left	-7.7
#11	0.633	0.633	left	0.0	1.464	1.395	right	-4.9

Note: ASI = (DL-NDL)/DL*100%; ASI: asymmetry index; D: dominant leg; NDL:non-dominant leg.

Table. Temperature and humidity information for four sessions.

	Temperature (℃)	Humidity (%)
Control group	18.3	55
Dynamic stretching group	19.6	53
Foam rolling group	20.3	54
Foam rolling combined with dynamic stretching group	19.8	50
Mean ± SD	19.5 ± 0.9	53 ± 2.2

Note. SD: standard deviation.
